# Prevalence and Characteristics of Extended-Spectrum-β-Lactamase-Producing and Carbapenemase-Producing *Enterobacteriaceae* from Freshwater Fish and Pork in Wet Markets of Hong Kong

**DOI:** 10.1128/mSphere.00107-20

**Published:** 2020-04-15

**Authors:** Dulmini Nanayakkara Sapugahawatte, Carmen Li, Chendi Zhu, Priyanga Dharmaratne, Kam Tak Wong, Norman Lo, Margaret Ip

**Affiliations:** a Department of Microbiology, Faculty of Medicine, Prince of Wales Hospital, The Chinese University of Hong Kong, Sha Tin, Hong Kong, SAR; b School of Biomedical Sciences, Faculty of Medicine, The Chinese University of Hong Kong, Sha Tin, Hong Kong, SAR; Antimicrobial Development Specialists, LLC

**Keywords:** ESBL, CPE, *Enterobacteriaceae*, freshwater fish, pigs, livestock, One Health, aquaculture, antimicrobial resistance

## Abstract

This study identified and characterized extended-spectrum-β-lactamase-producing *Enterobacteriaceae* (ESBL-E) and carbapenemase-producing *Enterobacteriaceae* (CPE) from farmed freshwater fish and pig offal procured from the wet markets across Hong Kong. During March 2018 to January 2019, 730 food animal samples, namely, 213 snakehead fish, 198 black carp, and 339 pig organs, were examined. ESBL-E and CPE were isolated from the homogenized samples plated on selective media and identified by matrix-assisted laser desorption ionization–time of flight mass spectrometry (MALDI-TOF-MS). All ESBL-E and CPE strains were tested for antimicrobial susceptibilities. ESBL-E and CPE gene groups were detected by multiplex PCR and *bla*_CTX-M-1/-2/-9_ group strains were Sanger sequenced for CTX-M types. All CPE isolates were whole-genome sequenced. Isolation of ESBL-E from pig small (52.4%) and large (50%) intestines and tongues (25.1%) was significantly (*P* < 0.05) more frequent than from snakehead (0.94%) and black carp (0.5%) fish. ESBL-E isolates (*n* = 171) revealed resistance rates of 16.3%, 29.8%, 35.6%, 53.2%, 55.0%, and 100% to piperacillin-tazobactam, amoxicillin-clavulanate, cefepime, gentamicin, ciprofloxacin, and ampicillin, respectively, whereas CPE (*n* = 28) were resistant to almost all the antibiotics tested except gentamicin, ciprofloxacin, and fosfomycin. The predominant ESBL gene groups in fishes and pig offals were *bla*_CTX_, where *bla*_CTX-M-55_ was the major subtype in the *bla*_CTX-M-1_ group (64.4% of isolates in the group). *bla*_CTX-M-14/-17_ was the major genotype in the *bla*_CTX-M-9_ group (32.2%). All CPE strains possessed *bla*_NDM_ genes. High rates of ESBL-E and CPE were identified in food animals from wet markets of Hong Kong, which may serve as a potential reservoir of antimicrobial-resistant genes and increase the challenges in tackling antimicrobial resistance beyond health care settings.

**IMPORTANCE** Extended-spectrum-β-lactamase-producing *Enterobacteriaceae* (ESBL-E) and carbapenemase-producing *Enterobacteriaceae* (CPE) are of global health importance, yet there is a paucity of surveillance studies on food animals in Hong Kong. Here, we report a high prevalence of ESBL-E (ranging from 0.5% to 52.4%) and CPE (0% to 9.9%) from various food animal samples procured from wet markets across Hong Kong. All CPE strains were characterized by whole-genome sequencing and possessed NDM-1 and -5 genes and other resistance determinants. Given the increased resistance profile of these strains, this study highlights the emerging threat of ESBL-E and CPE disseminated in farmed animals. Furthermore, our data enriched our understanding of antibiotic resistance reservoirs from a One Health perspective that can widely spread across various niches, beyond health care settings.

## Abstract (precis)

Extended-spectrum-β-lactamase-producing *Enterobacteriaceae* (ESBL-E) and carbapenemase-producing *Enterobacteriaceae* (CPE) are of global health importance, yet there is a paucity of surveillance studies on food animals in Hong Kong. Here, we report a high prevalence of ESBL-E (ranging from 0.5% to 52.4%) and CPE (0% to 9.9%) from various food animal samples procured from wet markets across Hong Kong. All CPE strains were characterized by whole-genome sequencing and possessed NDM-1 and -5 genes and other resistance determinants. Given the increased resistance profile of these strains, this study highlights the emerging threat of ESBL-E and CPE disseminated in farmed animals. Furthermore, our data enriched our understanding of antibiotic resistance reservoirs from a One Health perspective that can widely spread across various niches, beyond health care settings.

## INTRODUCTION

Extended-spectrum-β-lactamase-producing *Enterobacteriaceae* (ESBL-E) and carbapenemase-producing *Enterobacteriaceae* (CPE) are making a significant impact on antimicrobial resistance (AMR) due to their capability of horizontal gene transfer to other bacteria (1–4). The isolation rate of ESBL-E from humans soared in the past few decades, and the emergence of CPE around the globe has been described as foreshowing the end of the antibiotic era, with an urgent threat to public health worldwide (1–6). ESBL-E and CPE in food animals and their environment have been considered potential sources of resistant bacterial infections in the community (1). Increased consumption of antimicrobials, such as β-lactams, macrolides, aminoglycosides, polymyxins, and carbapenems, is observed on animal farms (1–3, 7, 8). Misuse of antibiotics in food animals, especially those used in the treatment of human infection, may lead to dissemination of highly mobile genetic elements in pathogenic bacteria that confer resistance to antimicrobials beyond the realm of health care settings to the community (1, 7, 8).

Animal-based food production is growing rapidly in Asia, with China as the world’s biggest producer of farmed ﬁsh in 2016, claiming over 60% (49.2 million tonnes) of the world’s production (80.0 million tonnes). The country has also been providing 54.5 million tonnes of pork around the world, supporting 50% of the global demand (9, 10). A consequence of the extensive use of antibiotics in aquaculture and pig farms could be the isolation of multidrug-resistant bacteria in these farms and their produce, which can eventually interfere with human gut commensals when animal food is consumed raw or undercooked.

Apart from therapeutic purposes, antibiotics are also administered to healthy animals for growth promotion or prevention of disease to improve production yields in regions round the globe (11). As a result, drug-resistant bacteria are frequently found in surveillance programs worldwide in farm animals, which led to hypotheses of potential spread of AMR bacteria to humans via the food chain and contaminated water (11, 12). There is limited understanding of the impact of the transmission of drug-resistant bacteria from animals to humans, yet there is a rising threat of AMR to global public health that requires action from societies and government sectors (10, 11). In addition to increased AMR awareness among professionals and consumers, surveillance systems for animal antibiotic use and antimicrobial resistance improve animal husbandry and are cornerstones to promote rational antibiotic use in animals (11).

In Hong Kong, fresh food products are often purchased from traditional wet markets. Currently, there are 97 public markets which are distributed across all districts in Hong Kong. They are run by the Food and Environmental Hygiene Department of Hong Kong SAR (13). Generally, the wet markets sell fresh meat, including poultry, beef, pork, etc., supplied from three licensed slaughterhouses (14) and include animal parts such as offal, head, tail, and feet, which are ingredients of the local cuisine. Live poultry are sold only at certain markets, with interventions in place to minimize zoonotic influenza transmission (15), while live marine fish and seafood may also be kept in “aquariums” before being sold.

Our present study sought to isolate and characterize ESBL-E and CPE from freshwater fish and pig gastrointestinal tract (GIT) organs procured from wet markets across Hong Kong during the period of April 2018 to January 2019 in order to understand the prevalence and significance of antimicrobial resistance in Hong Kong.

## RESULTS

### Prevalence of ESBL-E and CPE in freshwater fish and pig organs.

A total of 171 ESBL-E and 28 CPE strains were isolated in our study from 411 fish and 339 pig organs that were purchased in wet markets across all 18 districts of Hong Kong. Thirty-nine of the samples contained 2 ESBL-E species and/or CPE species. The percentages of food samples with ESBL-E isolated were 52.4% (44/84) of pig’s small intestine, 50% (32/64) of large intestine, 25.1% (43/171) of pig tongue, 0.9% (2/213) of snakehead fish, and 0.5% (1/198) of black carp samples. In addition to the low isolation rates of ESBL-E in fish, no CPE were identified from freshwater fish samples. However, 28 CPE strains were isolated from pig gastrointestinal tract (GIT) organs, with a prevalence rate of 8.15% (26/319). No ESBL-E or CPE were isolated from pigs’ livers, kidneys, tails, minced meat, or snout during our preliminary examination; thus, samples from these sites were not further investigated.

**FIG 1:**
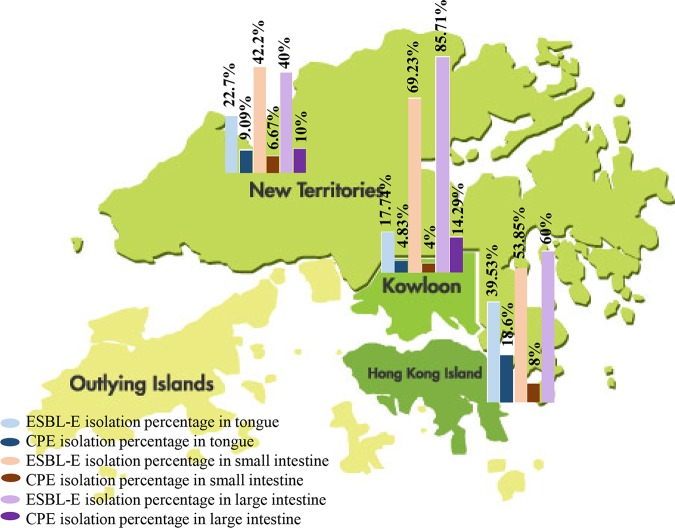
Prevalence of ESBL-E and CPE in pigs in the three geographical regions of Hong Kong.

The percentages of ESBL-E and CPE isolated from pig GIT organs from 3 regions, the New Territories, Kowloon, and Hong Kong Island, were compared (Fig. 1). ESBL-E and CPE were identified in 17.7% (ranging from 17.7% to 85.7%) and 4% of pig organs (ranging from 4% to 14.3%), respectively, across Hong Kong. Escherichia coli and Klebsiella pneumoniae were the major ESBL-E and CPE species in our study (Table 1). Isolation of ESBL-producing E. coli (ESBL-E. coli) was more frequent than ESBL-producing *Klebsiella* spp. (138 versus 33 isolates, respectively, where the latter included 29 Klebsiella pneumoniae, 3 Klebsiella variicola, and 1 Klebsiella oxytoca isolate). Four ESBL-E. coli strains were isolated from 2 snakehead fish, while 1 ESBL-producing K. pneumoniae (ESBL-K. pneumoniae) strain was isolated from 1 black carp, but no CPE were found. Twenty-eight CPE strains (25 E. coli and 3 K. pneumoniae strains) (Table 1) were isolated from 26 of 319 pig GIT organs (8.15%). Carbapenemase-producing K. pneumoniae (CP-K. pneumoniae) was isolated from 1.17% (2/171) and 1.56% (1/64) of tongues and large intestines, respectively, but none of the small intestine specimens were positive for CP-K. pneumoniae.

**TABLE 1:** Number of ESBL-E and CPE strains recovered from animal sources in wet markets of Hong Kong

Source	No. (%) of strains recovered					
	ESBL-E			CPE		
	Total (*n* = 171 [100%])	E. coli (*n* = 138 [80.7%])	*Klebsiella* spp. (*n* = 33 [19.3%])	Total (*n* = 28 [100%])	E. coli (*n* = 25 [89.28%])	Klebsiella pneumoniae (*n* = 3 [10.72%])
Snakehead fish	4 (2.35)	4 (2.35)				
Black carp	1 (0.58)		1 (0.58)^*a*^			
Pig tongue	55 (32.16)	46 (26.9)	9 (5.3)^*b*^	17 (9.94)	15 (53.58)	2 (7.14)
Pig small intestine	56 (32.75)	43 (25.15)	13 (7.6)^*c*^	5 (5.95)	5 (17.85)	
Pig large intestine	55 (32.16)	45 (26.3)	10 (5.82)	5 (9.38)	6 (17.85)	1 (3.58)

a The strain was K. pneumoniae.

b Included K. pneumoniae (*n* = 6) and *K. variicola* (*n* = 3).

c All 13 strains were K. pneumoniae.

### Antibiotic susceptibility test.

**FIG 2:**
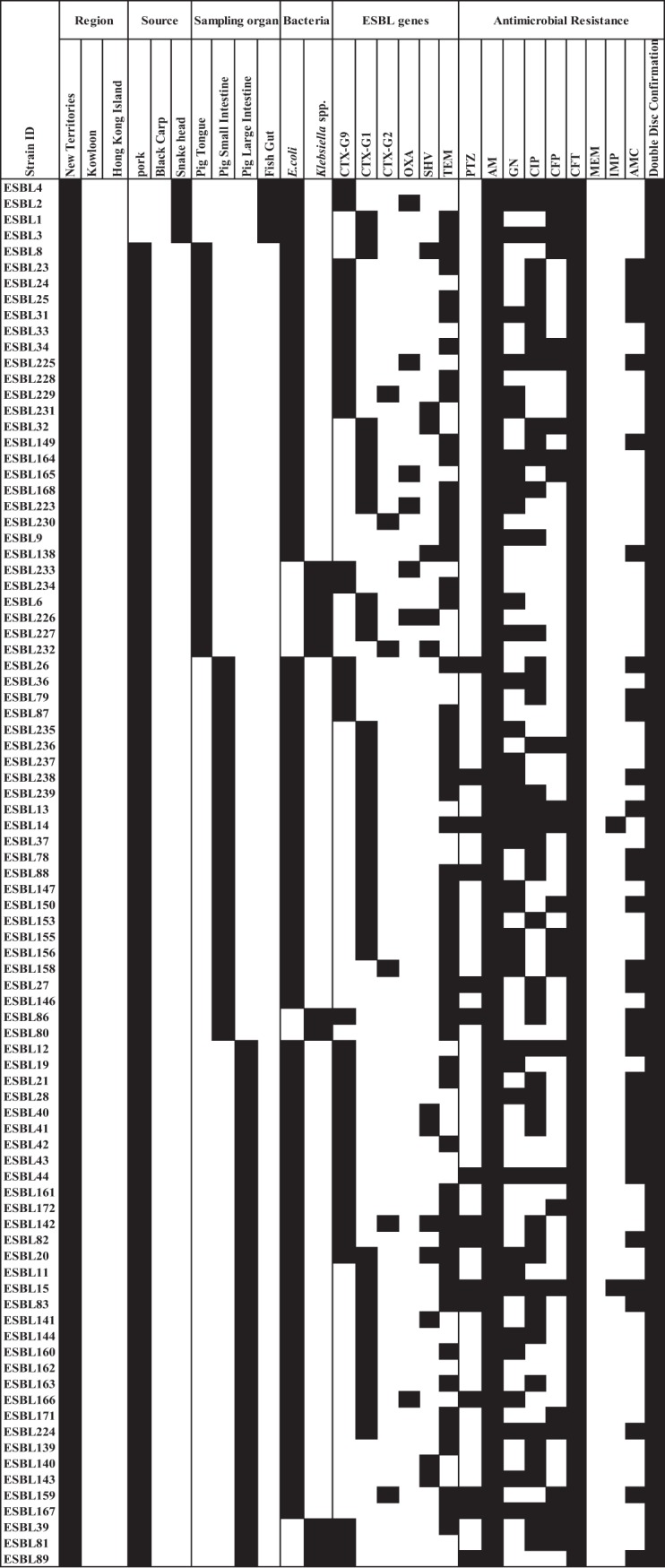

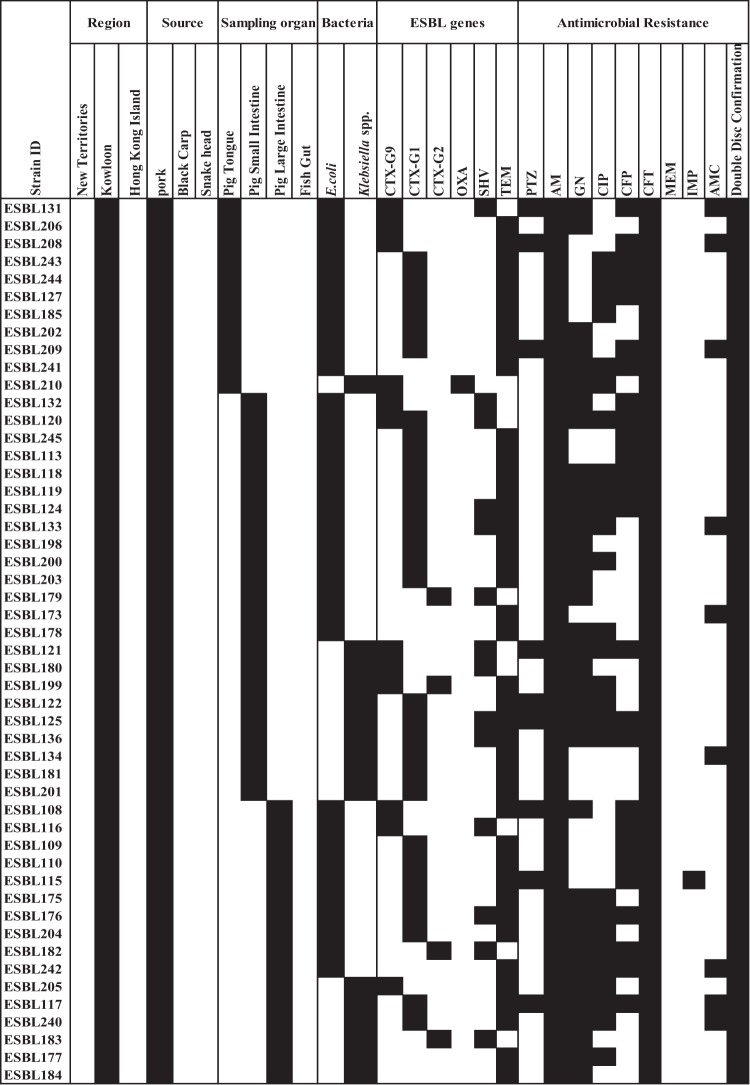

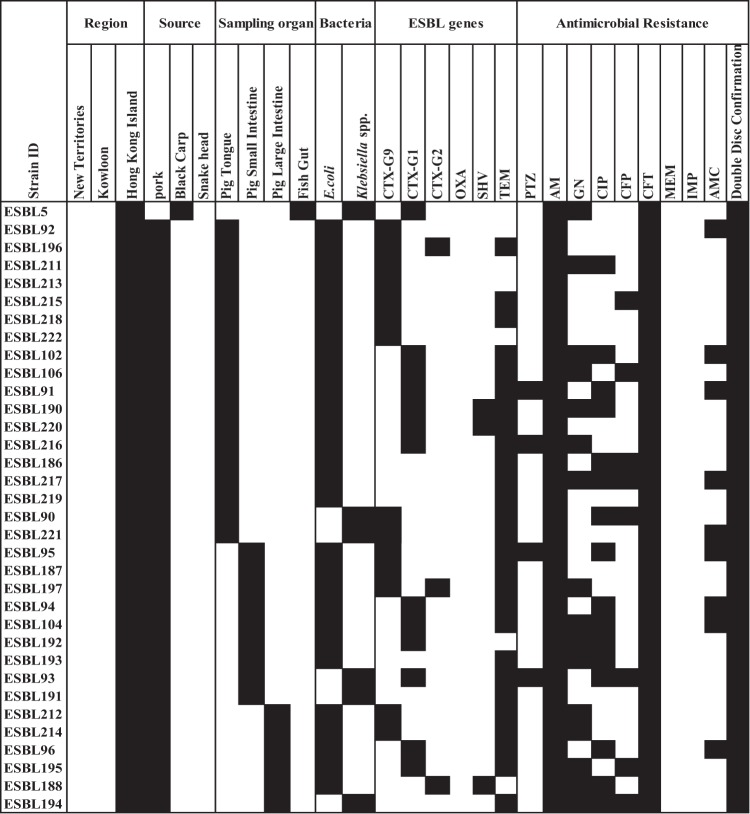
A schematic of ESBL-E isolated from pigs and fish in Hong Kong. Details of the ESBL-E isolates were segregated into 3 sourcing regions: (a) the New Territories, (b) Kowloon, and (c) Hong Kong Island. Details included the type of food source, sampling organ, bacterial species isolated, presence of ESBL genes (CTX group 1 [CTX-G1], CTX group 2 [CTX-G2], and CTX group 9 [CTX-G9]) and antibiogram (piperacillin-tazobactam [PTZ], ampicillin [AM], gentamicin [GN], ciprofloxacin [CIP], cefepime [CFP], ceftriaxone [CFT], meropenem [MEM], imipenem [IMP], amoxicillin-clavulanate [AMC], and cefotaxime [CTX]). Black squares indicate a feature present in the isolate and white squares denote features that are absent.

All 171 ESBL-E strains and 28 CPE strains were tested for 10 and 11 antibiotics, respectively. All ESBL-E strains were 100% and 98.3% susceptible to meropenem and imipenem, respectively (3 strains showed intermediate susceptibility by disk diffusion tests according to Clinical and Laboratory Standard Institute [CLSI] guidelines). The ESBL-E strains were resistant to piperacillin-tazobactam (16.3%), amoxicillin-clavulanate (29.8%), cefepime (35.6%), gentamicin (53.2%), ciprofloxacin (54.95%), ampicillin (100%), ceftriaxone (100%), and cefotaxime (100%) (Fig. 2).

**FIG 3:**
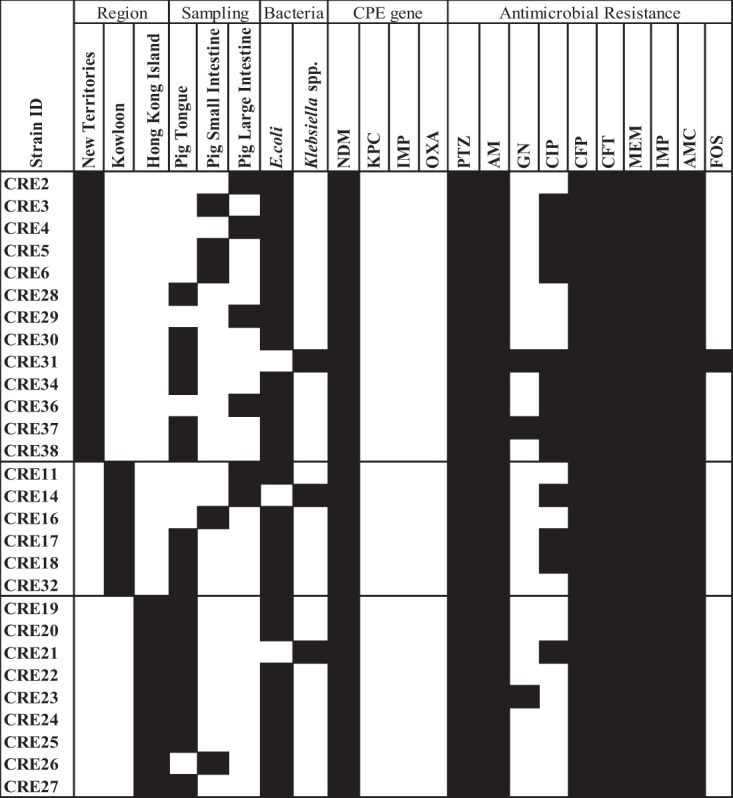
A graphic representation of CPE isolated from pigs. Details of CPE showing the region of sample collection (New Territories, Kowloon, and Hong Kong Island), sampling organ (pig tongue, pig small intestine, and pig large intestine), isolated bacterial species (Escherichia coli, *Klebsiella* spp.), presence of CPE gene groups (black squares), and antibiogram (piperacillin-tazobactam [PTZ], ampicillin [AM], gentamicin [GN], ciprofloxacin [CIP], cefepime [CFP], ceftriaxone [CFT], meropenem [MEM], imipenem [IMP], amoxicillin-clavulanate [AMC], and cefotaxime [CTX], and fosfomycin [FOS]). Black squares indicate a feature present in the isolate and white squares denote features that are absent.

Similarly to ESBL-E, all our 28 CPE strains were resistant to piperacillin-tazobactam, ampicillin, cefepime, ceftriaxone, meropenem, imipenem, amoxicillin-clavulanate, and cefotaxime, whereas 3.5%, 10.7%, and 46.4% of the CPE strains were resistant to fosfomycin, gentamicin, and ciprofloxacin, respectively (Fig. 3).

### Molecular analysis of ESBL-E and CPE strains.

ESBL gene groups were determined for our 171 ESBL-E strains (*bla*_OXA_, *bla*_SHV_, *bla*_TEM_, and *bla*_CTX-M_ groups 1, 2, and 9). The most prevalent group was CTX-M (91.8% [157/171]), followed by TEM (71.9% [123/171]), SHV (16.9% [29/171]), and OXA (4.6% [8/171]). Among *bla*_CTX-M_ groups, PCR results revealed that 7.65% (13/171), 36% (62/171), and 47.9% (82/171) belonged to *bla*_CTX-M_ groups 1, 2, and 9, respectively. Two E. coli strains (1.1%) carried 2 *bla*_CTX_ genes (*bla*_CTX-M-1_ and *bla*_CTX-M-9_ groups) and 5 E. coli strains (2.9%) carried genes from *bla*_CTX-M-2_ and *bla*_CTX-M-9_ groups. *bla*_CTX-M-8_ and *bla*_CTX-M-25_ groups were not detected among the isolates (Fig. 2). All strains positive for *bla*_CTX-M_ groups 1, 2, and 9 were Sanger sequenced to delineate the possible β-lactamases as listed in Table 2. *bla*_CTX-M-55_ was the major subtype in the *bla*_CTX-M-1_ group, accounting for 64.4% (52/82) of strains, while the remaining subtypes included *bla*_CTX-M-69_, *bla*_CTX-M-3_, and *bla*_CTX-M-226_. On the other hand, *bla*_CTX-M-14/-17_ was the major subtype (32.2% [20/62]) in the *bla*_CTX-M-9_ group. Further elucidation of the subtypes of TEM, SHV, and OXA genes were not performed; thus, there is a possibility that non-ESBL TEM/SHV genes were included. However, most strains were already confirmed to be ESBL-E and to possess CTX-M genes.

**TABLE 2:** β-Lactamases detected in ESBL-E strains recovered from animal sources in wet markets of Hong Kong

Allele group	No. of positive strains^*a*^	Enzyme(s) detected	
		Type	No. of strains
*bla*_CTX-G1_	82	CTX-M-139/-183	1
		CTX-M-15^*b*^/-28/-139/-156/-163/-186/-194/-216/-224	2
		CTX-M-15^*b*^/-28/-139/-163/-186/-194/-216/-224	1
		CTX-M-15^*b*^/-28/-163/-186/-194/-216/-224	1
		CTX-M-226	1
		CTX-M-3/-22/-66/-162/-211	12
		CTX-M-55^*b*^/-69/-79/-164/-226	43
		CTX-M-55^*b*^/-79/-164/-226	9
		CTX-M-69	12
*bla*_CTX-G2_	13		ND^*c*^
*bla*_CTX-G9_	62	CTX-M-121	1
		CTX-M-122	1
		CTX-M-130	4
		CTX-M-14^*d*^	2
		CTX-M-14^*d*^/-17	20
		CTX-M-17	5
		CTX-M-24	8
		CTX-M-24/-196	1
		CTX-M-27/-174	8
		CTX-M-65	11
		CTX-M-98	1

a Numbers of positive samples do not add up to a total of 171, because isolates may possess several *bla* genes.

b CTX-M-55 and CTX-M-15 were the most predominant in the *bla*_CTX-M-1_ group.

c ND, not done.

d CTX-M-14 was the most predominant in the *bla*_CTX-M-9_ group.

**FIG 4:**
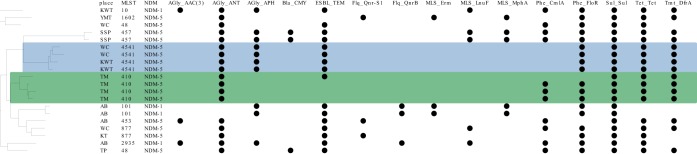
Pangenome tree of carbapenem-producing E. coli. The pangenome tree indicates the place of purchase, MLST of the strains, NDM groups, and antimicrobial resistance genes in the genome. Antimicrobial resistance genes positive among the strains are shown with black dots.

Molecular analysis for our 28 CPE showed all the strains carried the *bla*_NDM_ gene. Twenty-three of 28 CPE isolates were whole-genome sequenced, including 20 E. coli and 3 K. pneumoniae strains. Sixteen of 20 CP-E. coli carried the *bla*_NDM-5_ gene, with sequence types (ST) 410 (*n* = 4), ST4541 (*n* = 4), ST457, ST877 (2 strains for each STs), and single strains of other ST types (Fig. 4). The remaining 4 strains showed the *bla*_NDM-1_ gene and were ST101 (*n* = 2), ST10 (*n* = 1), and ST2935 (*n* = 1). All except two CPE strains had their *bla*_NDM_ genes located on the IncX3 plasmid (plasmid size approximately 46,161 bp). Genes inferring aminoglycoside resistance (*ant* gene), chloramphenicol resistance (*floR* gene), sulfonamide resistance (*sul* gene), tetracycline resistance, and trimethoprim resistance (*dfrA* gene) were observed in more than 75% (15/20) of the strains.

The genetic characteristics of CP-K. pneumoniae are described in Table 3. One ST127 strain had the *bla*_NDM-5_ gene on an IncX3 plasmid together with *bla*_SHV_ and *bla*_TEM_. The other 2 strains (serotypes K154:O2 and K152:OL102) showed *bla*_NDM-1_ genes on IncX3 plasmids as well as the presence of *bla*_SHV_. Similarly to ESBL*-*E. coli, genes inferring trimethoprim (*dfrA* gene), sulfonamide (*sul* gene), and aminoglycoside (*aac* genes) resistance were found. In addition, fluoroquinolone resistance gene *oqxAB* was found in all 3 K. pneumoniae strains. The presence of *oqxAB* was associated with low to intermediate resistance to quinoxalines, quinolones, tigecycline, nitrofurantoin, and several detergents and disinfectants (16). One of the CP-K. pneumoniae strains also contained the *aac(6′)-Ib-cr* gene with associated ciprofloxacin resistance in accordance with previous reports (17, 18). The KP23 strain was found to carry the *fosA* gene in the whole-genome analysis, which may explain the phenotypic resistance to fosfomycin.

**TABLE 3:** Genetic characteristics of 3 Klebsiella pneumoniae strains

Strain	ST	Serotype	NDM	NDM plasmid	Other AMR genes^*a*^	Virulence factors^*a*^
KP7	199	K154:O2	NDM-1	IncX3	*bla*_SHV_, *oqxAB*, *qnrS*, *drfA*, *aadA*, *sul*, *tet*	
KP14	Not found	K152:OL102	NDM-1	IncX3	*bla*_SHV_, *bla*_TEM_, *oqxAB*, *qnrB*, *drfA*, *aadA*, *sul*, *aac(3)-IId*, *floR*, *tet*	*ybt*, *iuc*, *fyuA*
KP23	127	K30:O2	NDM-5	IncX3	*bla*_SHV_, *bla*_TEM_, *oqxAB*, *qnrB*, *drfA*, *aadA*, *sul*, *aac(3)-IId*, *aac(6')-Ib-cr*, *aph(3')-Ia*, *floR*, *tet*, *fosA*, *mphA*, *aph(6)-Id*, *aph(3′')-Ib*	*ybt*, *irp*, *iuc*, *fyuA*

a BLAST cutoff of AMR gene and virulence factor coverage, 95%; identity, 95%; depth of *de novo* assembly contigs, 5.

## DISCUSSION

There are limited data on the surveillance of ESBL-E and CPE in aquaculture and food animals, albeit similar data have been extensively reported in health care settings (19). We thus conducted this study to investigate the distribution and characteristics of ESBL-E and CPE in our food chain. This territory-wide surveillance study not only provides an update to the burden of ESBL-E and CPE in food products from wet markets but also highlights the possible exposure of these resistant bacteria in the community.

This is the first report on ESBL-E and CPE from freshwater fish sampled in Hong Kong. The ESBL-E rate for freshwater fish was low (3/411 [0.72%]) with *bla*_CTX_ gene groups being the predominant ESBL gene groups. The low isolation rate may be due to the recent restriction in the use of third-generation cephalosporins in Hong Kong aquaculture (3). Our isolation rates were much lower than from studies performed in Saudi Arabia (27.2% [110/405], *bla*_CTX-M_ predominance) (6), Egypt (5.1% [14/274]), and China (1.5% [3/218]) (20, 21), and ESBL genes were less heterogeneous than in another Chinese study (*bla*_TEM_, *bla*_SHV_, *bla*_CTX-M_, and *bla*_LEN_ in ESBL-E. coli) (18). The predominant ESBL gene identified from fish sampled in Saudi Arabia was *bla*_CTX-M_, which was detected in all tilapias imported from Thailand (6). *Bla*_CTX-M_ was also observed in all carfu fish, 60% of milkfish, 52.3% of catfish, and 34.8% of tilapia, which were all imported from India (6). The original source of our fish is believed to be local as well as from Guangdong Province (southern part of China), while enquiries with the hawkers revealed also importation from Southeast Asia, such as Vietnam. Hence, CTX-M is still the most prevalent ESBL type among freshwater fish in Southeast Asia.

ESBL-E and CPE isolation rates were much higher in pigs than in freshwater fish in Hong Kong. Our isolation rates were higher than those reported in Thailand (36.7% of 588 pig farms) (22), Portugal (24.6% of 65 pig fecal samples; *bla*_CTX-M-1_ predominance) (23), and Cameroon (11.26% [8/71] of pigs) (24). A study in Denmark identified ESBL-producing E. coli in 79% (15/19) of pig farms with high consumption of cephalosporin compared to 20% (4/20) of the pig farms with no consumption (25). The former showed a predominance of *bla*_CTX-M_ followed by *bla*_SHV_ genes (25). On the other hand, our results were noticeably lower than a previous surveillance study during 2008 to 2010 from pig feces (63.6% [136/214]) in Hong Kong (26). This discrepancy might be due to the differences in sample types and processing methods. It may also be due to the recently introduced food-related initiatives under the Hong Kong Strategy and Action Plan on antimicrobial resistance (AMR) and licensing control of livestock keeping, regulating the feeding of drugs and chemicals to food animals in 2017 (2, 27). Under this regulation, seven chemicals, including two antibiotics (avoparcin, clenbuterol, chloramphenicol, dienestrol, diethylstilbestrol, hexestrol, and salbutamol) are prohibited for use in food animals. Moreover, chemicals, including 36 antibiotics, have restricted usage on animals to address the concern of proper antibiotic usage and non-exceedance of drug residue levels for food safety purposes and AMR issues in Hong Kong (2, 3, 28).

Similarly to a Turkish study on fish of the Eastern Mediterranean (29), no CPE were detected in our freshwater fish. However, scanty reports of CPE in seafood were published during 2011 to 2016, with a *bla*_VIM-1_-expressing E. coli (ST10) isolated from a Venus clam (Ruditapes philippinarum) in the Mediterranean Sea (Italy) (30) and carbapenem-resistant *Enterobacteriaceae* in 0.6% (8/1,328) of the seafood samples from a Canadian study, where all the samples were imported from Southeast Asian countries, specifically, Vietnam and Bangladesh (31).

This is the first study in Hong Kong to report NDM-1 and NDM-5 in pig offal from local farms. However, *bla*_NDM-1_ and *bla*_NDM-5_ were also reported in China, where the former subtype was found in E. coli from diseased pigs lung samples (0.89% [3/334]) in 2013 in Guangdong Province (32) and the latter subtype from imported pigs in Hong Kong originating from Guangdong, Henan, and Hunan provinces during 2015 and 2017, where the carbapenem-resistant *Enterobacteriaceae* (CRE) isolation rate was 0.7% from 856 samples (33). The CRE isolation rate was as high as 60% (18/30) in environmental samples collected from pig farms in the United States (34). The increased isolation of CRE urges an immediate and sustainable plan of action to overcome the dissemination of AMR in all sectors, including agricultural, veterinary, and public health sectors, worldwide.

There is a substantial number of overseas studies on the efficacy and cost efficiency of interventions to reduce AMR (35, 36). However, local surveillance studies are limited, and it is important to investigate the possible effect and feasibility of new measures. From surveillance of AMR, the knowledge of the associated bacteria and molecular elements are important in our aim to control the multidrug-resistant (MDR) Gram-negative pathogenic infection burden in the veterinary and public health sectors (2, 3, 10, 11). Thus, our results will be valuable as a baseline to guide interventions in reducing AMR in agriculture, farms, and the community.

In conclusion, our study is the ﬁrst to demonstrate the presence of ESBL genes in ﬁsh purchased from Hong Kong wet markets. Efforts should be made worldwide to closely monitor and introduce control of antibiotic resistance in aquaculture as well as pig farms. Our results depict a major reservoir of resistance genes that extend beyond our health care environments and threaten our dwindling options of effective antibiotics in future human medicine. Further epidemiological studies and detailed analyses of the mobile genetic elements encoding these genes should also be conducted to assess the full extent of zoonotic transmission and dissemination of these AMR genes between animal and humans.

## MATERIALS AND METHODS

### Sample collection.

Food animal samples were purchased from 18 wet markets to include one each from a district and to represent three geographical regions (New Territories, Kowloon, and Hong Kong Island) of the city. Food animals, including gut samples of 213 snakehead fish (*Channa* spp.) and 198 black carp (*Mylopharyngodon* spp.) and 339 pig organs (171 tongues, 84 small intestines, 64 large intestines, 9 minced meat samples, 4 tails, 3 livers, 3 kidneys, and 1 snout). They were obtained between April 2018 and January 2019. Samples were transported and stored at 4°C after purchase and were processed within 24 h.

### Isolation of ESBL-E and CPE.

Deep tissues, where applicable in the food sample, were dissected using sterile equipment to avoid handling and environmental contamination (37). A small piece of tissue was transferred to 3 ml of normal saline and homogenized prior to seeding 10 μl of the homogenized sample on Chromid ESBL agar (bioMérieux, France), which was then incubated at 37°C for 18 to 24 h (38). Presumptive *Enterobacteriaceae* colonies were selected from each plate and identified by matrix-assisted laser desorption ionization–time of flight mass spectrometry (MALDI-TOF-MS) (Bruker Daltonics, Inc., Germany) followed by phenotypic confirmation via double disc synergy test according to CLSI guidelines (39). All confirmed ESBL-E strains were saved at −80°C in 10% (vol/vol) glycerol-brain heart infusion (BHI) broth (Oxoid, UK) for further analysis.

CPE were isolated by transferring 2 ml of homogenized normal saline suspension (mentioned above) to sterile tubes containing 8 ml of Trypticase soy broth (TSB) (Oxoid, UK) enriched with 1 mg/liter meropenem (Oxoid, UK) prior to incubation at 37°C for 18 to 24 h. Ten microliters of incubated broth was seeded on CARBA SMART agar (bioMérieux, France) and incubated at 37°C for 18 to 24 h (38, 40). Presumptive *Enterobacteriaceae* colonies were selected from each plate and identified by MALDI-TOF-MS followed by phenotypic confirmation using a carbapenem inactivation method (CIM) as described previously (41). All confirmed CPE strains were saved at −80°C in 10% (vol/vol) glycerol-BHI broth (Oxoid, UK) for further analysis.

### Antibiotic susceptibility testing.

Antimicrobial susceptibility testing was performed using the agar disk diffusion method according to CLSI recommendations (42). The antimicrobial disks tested were piperacillin-tazobactam (PTZ; 100/10 μg), ampicillin (AM; 10 μg), gentamicin (GM; 10 μg), ciprofloxacin (CIP; 5 μg), cefepime (CFP; 30 μg), ceftriaxone (CFT; 30 μg), meropenem (MEM; 10 μg), imipenem (IMP; 10 μg), amoxicillin-clavulanate (AMC; 20/10 μg), and cefotaxime (CTX; 30 μg) for ESBL-E and additionally fosfomycin (FOS; 200 μg) for CPE strains. Escherichia coli ATCC 25922 was used as a control (42).

### Screening for ESBL-producing isolates.

The combination disk method was used to confirm ESBL-E strains. In brief, pairs of disks containing cefotaxime (30 μg) and ceftazidime (30 μg) were used with and without clavulanic acid (10 μg) on the same inoculated plate containing Muller-Hinton agar (Oxoid, UK). A positive test result was defined as a ≥5-mm increase in the zone diameter compared to that of a disk without clavulanic acid (42).

### Molecular characterization of ESBL-E and CPE.

**(i) DNA extraction, PCR ampliﬁcation, and amplicon sequencing.** Two to four bacterial colonies were emulsified in 200 μl of distilled water, heated at 95°C for 15 min, and centrifuged at 16,000 × *g* for 5 min (43). The supernatants were directly used as the template DNA and stored at −20°C until use. All confirmed ESBL-E strains were screened using multiplex PCRs as previously described for the detection of *bla*_CTX-M_ genotype groups 1, 2, and 9, *bla*_TEM_, *bla*_SHV_, *bla*_OXA_, and genes encoding carbapenemase groups, *bla*_KPC_, *bla*_NDM_, *bla*_OXA_, and *bla*_IMP_ (44, 45). All ESBL-E positive for *bla*_CTX-M_ genotype groups 1, 2, and 9 were further subjected to another set of PCRs for the detection of gene encoding the CTX-M enzyme by Sanger sequencing as described previously (45).

**(ii) Whole-genome sequencing of CPE.** DNA of CPE strains was extracted using a Wizard Genomic DNA purification kit (Promega, USA) according to the manufacturer’s protocol, followed by library preparation via a Riptide high-throughput rapid DNA library preparation kit (iGenomx, USA) according to manufacturer’s instruction. Genomes were sequenced by NextSeq mid-output 500 obtaining paired-end reads at 150 bp (Illumina, USA). Sequence reads were demultiplexed according to the manufacturer’s instructions for the library preparation kit prior to our genome assembly pipeline, as previously described (46). Briefly, *de novo* assembly of the sequence reads was generated by SPAdes (3.10.1) (47), where contigs with a depth of <5 and length of <500 bp were filtered. Resistant gene profiles and plasmid replicons were acquired by blasting and read mapping to ResFinder (version 2019-02-20) and PlasmidFinder (version 2018-11-20) (48, 49). Virulence factors were identified using VFDB (50). PubMLST database was used for multilocus sequence typing (MLST) (51). A pangenome tree was constructed by Roary and visualized by ETE (52, 53). The genetic environment of the carbapenemase gene was scaffolded two ways: (i) raw reads were mapped to reference plasmid by Bowtie 2 (2.3.4.1); (ii) contigs from the *de novo* assembly were aligned to a reference plasmid (18). If the coverage of both methods was >80%, this reference was treated as a draft plasmid.
